# Low Mg content on Ti-Nb-Sn alloy when in contact with eBMMSCs promotes improvement of its biological functions

**DOI:** 10.1007/s10856-021-06620-9

**Published:** 2021-12-04

**Authors:** Carolina da Silva Dias, Mariana Correa Rossi, Emanuel V. P. Apolonio, Gustavo dos Santos Rosa, João Pedro Hübbe Pfeifer, Carlos Alberto Hussni, Marcos Jun Watanabe, Ana Liz Garcia Alves

**Affiliations:** Department of Veterinary Surgery and Anesthesiology, São Paulo State University - Júlio de Mesquita Filho Unesp Prof. Doutor Walter Mauricio Correa St., n/n, Botucatu, SP ZIP- 18618-681 Brazil

## Abstract

Magnesium is a metal used in the composition of titanium alloys and imparts porosity. Due to its osteoconductive, biocompatible and biodegradable characteristics, its application in the development of biomedical materials has become attractive. This study aimed to evaluate the influence of magnesium present in porous Ti-Nb-Sn alloys, which have a low elastic modulus in adhesive, osteogenic properties and the amount of reactive intracellular oxygen species released in mesenchymal stem cells derived from bone marrow equine bone (eBMMSCs). Mechanical properties of the alloy, such as hardness, compressive strength and elastic modulus, were analyzed, as well as surface morphological characteristics through scanning electron microscopy. The evaluation of magnesium ion release was performed by atomic force spectroscopy. The biological characteristics of the alloy, when in contact with the alloy surface and with the culture medium conditioned with the alloy, were studied by SEM and optical microscopy. Confirmation of osteogenic differentiation by alizarin red and detection of ROS using a Muse® Oxidative Stress Kit based on dihydroetide (DHE). The alloy showed an elastic modulus close to cortical bone values. The hardness was close to commercial Ti grade 2, and the compressive strength was greater than the value of cortical bone. The eBMMSCs adhered to the surface of the alloy during the experimental time. Osteogenic differentiation was observed with the treatment of eBMMMSCs with conditioned medium. The eBMMSCs treated with conditioned medium decreased ROS production, indicating a possible antioxidant defense potential of magnesium release.

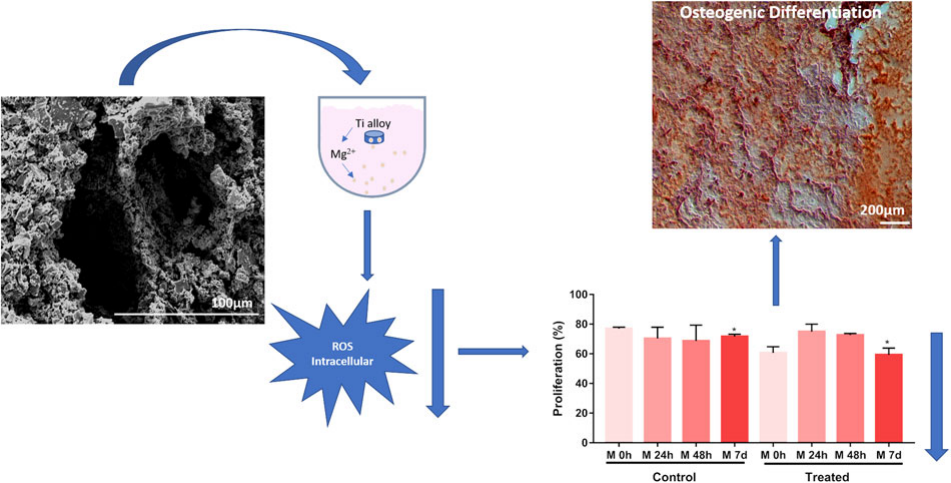

## Introduction

Niobium (Nb) and Tin (Sn) alloy elements are being used to obtain a promising titanium alloy (Ti-Nb-Sn) to be applied in orthopedic materials and have been widely studied [1–4]. The metals most used in commercial biomedical devices are stainless steel, cobalt-chromium alloys (CoCr) and titanium (Ti) associated with aluminum (Al) and vanadium (V) (Ti-6Al-4V). The most commonly used stainless-steel alloy is 316 L, which is composed mainly of chromium (Cr), nickel (Ni) and molybdenum (Mo) [5]. However, these alloys have raised concerns about the potential risks caused by metal ions released by corrosion caused by the body’s saline environment, which may accumulate in tissues, especially metals known as carcinogens, such as Cr, Co and Ni [6]. Studies carried out on bone marrow stromal cells cultured with metals found in Co-Cr-Mo and 316 L alloys resulted in hexavalent Cr as a significant cytotoxic element and Co, Mo and Ni as moderately cytotoxic elements [7]. Other studies revealed that Ti-6Al-4V alloy, the vanadium element, was the most toxic element, further suggesting the existence of a synergistic interaction between titanium (Ti), aluminum (Al) and vanadium (V), where Ti-6Al-4V produced toxic effects in concentrations in which the individual elements did not produce [7]. However, the biological behavior of metals can show the importance of the composition of implant biomaterials, which must be carefully selected to avoid or minimize adverse reactions [8]. Commercial Ti alloys are the most suitable for orthopedic applications due to their good resistance to corrosion and mechanics [9–11]. Ti, niobium (Nb), tantalum (Ta) and tin (Sn) are believed to be nontoxic metals with good biocompatibility [10]. Compared to Ta, Ti and Nb are inexpensive materials and exhibit better biocompatibility [12]. In addition, these alloys are formed by β-stabilizing elements (Nb and Sn), which provide mechanical characteristics more similar to those of bone tissue [13]. The Ti-Nb-Sn alloy used in this study was made with the addition of magnesium powder (Mg) because its ability to increase porosity and roughness from its partial evaporation can promote good biocompatibility, contributing to adhesion and osteogenic differentiation [14]. The central objective of this study was to investigate the osteogenic differentiation ability of mesenchymal stem cells derived from equine bone marrow (eBMMSCs) when in indirect contact with a promising porous Ti alloy (Ti-Nb-Sn/Mg) and the potential antioxidant activity by decreasing the release of intracellular reactive oxygen species (ROS).

## Materials and methods

### Biomaterial Synthesis

The Ti-Nb-Sn/Mg (60 wt% Ti, 34 wt% Nb and 6 wt% Sn) alloy was obtained by powder metallurgy. First, the TiH (ASTM F67), NbH (99.99%) and atomized Sn (99.50%) powders supplied by CBMM (Araxá-MG-Brazil) and Metalpó (São Paulo-Brazil) companies and Mg (3 wt%) were mixed in a high-energy planetary mill (FRITZCH-model Pulverisette 5) by the blended elemental method. After 40 min of mixing, they were dried under vacuum and compacted uniaxially in a 1 cm² matrix at a pressure of 100 MPa. Sintering was carried out in two stages: (1) at 400 °C for 1 h and then 800 °C for 2 h of heating. The heat process was performed in a high vacuum resistive furnace (COMBUSTOL—model Tubular furnace) coupled to a mechanical pump (Edwards) and diffuser (Edwards), which provided a pressure less than 10^−2^ Pa. Before heating, the furnace chamber was replaced with argon and evacuated twice.

For biological tests, samples were sectioned with dimensions of 12 mm in diameter and 2.0 mm in thickness and then washed with a 1-hour ultrasonic bath containing isopropyl alcohol and acetone (1:1). Finally, they were dried and autoclaved at 120 °C for 1 h. Sterilization was performed for 1 h to ensure possible contamination.

### Microstructure and Mechanical properties of TiNbSn/Mg

The microstructure of the Ti-Nb-Sn/Mg alloy was examined by scanning electron microscopy (SEM) (from FEI, Quanta 200) coupled with energy-dispersive X-ray spectroscopy (EDS) (from Oxford, 51 -XMX1119). EDS analysis was performed to evaluate the presence of Ti, Nb, Sn, C and Mg elements. EDS elemental maps were also obtained to display the distribution of chemical elements such as Ti, Nb, Sn, Mg and C. The hardness was measured using a BECLA durometer by the Rockwell method (BECLA), with a spherical steel indenter of 1/16” diameter for 30 s of indententation according to ASTM E92 17 and ASTM E384 18 standards. The hardness and Rmax values were based on the average of five measurements. The elastic modulus, E (GPa), was obtained using ATCP® Sonelastic impulse excitation equipment. The values were based on the average of six measurements. Compressive tests were performed in a SHIMADZU-AG-X plus, 100 kN, universal testing machine. The values were also based on the average of five measurements.

### Magnesium ion release

First, the materials (*n* = 3) were cleaned and added to sterile bottles containing 50 ml of Ringer Hartmann solution. The bottles together with the samples were incubated at 37 °C. Then, the liquids were removed, and ionic quantification took place by atomic absorption spectroscopy of hardened plasma coupling (ICP-OES) (Varian 715-S). The wavelength applied to determine the content of ions released was 279.8 nm (Mg).

### Cell culture

Cell culture was performed by isolating cells originating from bone marrow (BM) in horses. The present work was approved by the ethics committee, according to protocol CEUA 0235/2018. The eBMMSCs were initially cultured with conventional culture medium (Gibco-Thermo Fisher) supplemented with 10% fetal bovine serum (FBS), 100 U/ml penicillin and 100 mg/ml streptomycin (Gibco-Thermo Fisher). They were kept incubated in an atmosphere of 5% CO_2_ at 37 °C. The medium was changed every 3 days until reaching confluence for later use.

### Direct contact adhesion test

A qualitative assay was performed using eBMMSCs (1 × 10^5^) plated directly on the surface of the biomaterials (*n* = 3 discs), placed on a 24-well (15.6 mm) culture plate (one disk per well) and incubated for 48 h with conventional culture medium (without FBS) for morphological and adhesive evaluation compared to other materials in the literature. After 48 h, the cells were fixed with glutaraldehyde (2.5%) in 0.1 M phosphate buffer (pH 7.3) and kept for another 24 h in the refrigerator. Subsequently, they were dehydrated in aqueous solutions with increasing ethanol concentration [7.5% (2 × 10 min), 15% (2 × 10 min), 30% (2 × 10 min), 50% (2 × 10 min), 70% (3 × 15 min), 90% (2 × 15 min) and 100% (2 × 10 min)] and subjected to drying above the critical point in CO_2_. The specimens were observed using a scanning electron microscope (SEM) (FEI, Quanta 200) after metallization by a gold layer.

### Osteogenic differentiation assay by indirect contact

eBMMSCs were seeded in 6-well (1 cm²) culture plates (*n* = 10, 5 wells for the control group and 5 wells for the treated group). Initially, 1 × 10^6^ cells/well were plated, and all wells received conventional culture medium (2 mL). After semiconfluence, the conventional medium was replaced with osteogenic differentiation and enriched with ascorbic acid (50 μg/ml), ß-glycerophosphate (10 μM) and dexamethasone (0.1 μM) (Sigma-Aldrich Brasil Ltd). This differentiation medium was previously conditioned with the biomaterial (conditioned osteogenic medium) for 24 h before being used to assess the possible release of harmful particles that could influence the osteogenic differentiation process. This conditioned osteogenic medium with the inductors and the biomaterial were stored in the refrigerator throughout the experiment. The conditioned medium with the alloy was carried out according to the ISO 10993-5 standard. The control group, however, received only conventional culture medium during the entire period. Both remained incubated at 37 °C and 5% CO_2_, with the medium changing every 3 days. The influence of the conditioned osteogenic medium was assessed at the following times: 0 h, 3 days, 10 days and 15 days. At each time point, the cultures were fixed in 70% alcohol and stained with Alizarin Red, according to the experimental protocol, to validate the process of differentiation from calcium deposition by the extracellular matrix for optical microscopy (inverted phase contrast microscope, Axiovert 40, Zeiss). For quantitative analysis of Alkaline Phosphatase (ALP), the samples were placed into 96-well plates to measure the optical density (OD) at λ = 450 nm in a microplate reader (Highland Park, Winooski, VT, USA) Gen5 software.

### Release of reactive oxygen species (ROS)

The objective of this assay was to quantify the intracellular release of ROS by eBMMSCs treated with conditioned medium with the biomaterial. A hypoxic environment is closely linked with proliferation, adhesion, cell death and consequent tissue repair. The cells (2 × 10^5^ cells/well) were seeded in 2 plates of 6 wells (*n* = 12): 6 wells for the control group and another 6 wells for the treated group. Initially, all of them were treated with conventional medium (2 mL) until they reached semiconfluence. Then, the cells started to be treated with conditioned medium with the biomaterial according to ISO 10993-5. Briefly, the biomaterials (*n* = 5) were added to tubes (15 mL) containing 5 mL of conventional culture medium (FBS free). They were incubated under the same conditions as the plated cells 24 h before starting the experiment. In addition, the cells were treated with the conditioned medium for 4 different times, namely, 0 h, 24 h, 48 h and 7 days. Intracellular ROS were detected using a Muse® Oxidative Stress Kit based on dihydroetide (DHE). The assay allowed us to distinguish two populations of living cells: eBMMSCs ROS (−) and eBMMSCs ROS (+) exhibiting a high ROS content. The working solution was prepared by diluting the oxidative stress reagent using a buffer solution (Assay Buffer) at a proportion of 1:100, forming the intermediate solution. Then, it was diluted again in assay buffer at a 1:80 ratio, resulting in the working solution. For the analysis, the cells were scraped and centrifuged in 1 mL of assay buffer for 10 min at 2000 rpm. The cell pellet was diluted (1 × 10^6^ cells in 1 mL of assay buffer). Finally, 190 μL of working solution with 10 μL of cells was added and incubated for 30 min at 37 °C. Ultimately, the reading was performed on the equipment.

### Statistical analysis

Experimental data such as hardness, elastic modulus, mineralization quantification, eBMMSCs proliferation and the number of eBMMSCs ROS (+) and (−) are reported as the mean ± standard deviation (SD). The SD was also used as an error bar in the figures and table. For double and multiple comparisons, *t* tests and one-way ANOVA were used. Differences were considered statistically significant when the *P* value was <0.05. For the statistical analyses, plot curves and bar graphs were generated using GraphPad Prism v.7 (GraphPad, San Diego, CA, USA) and OriginPro 8.5 software.

## Results

### Morphology and mechanical properties of TiNbSn/Mg alloy

**Fig. 1:**
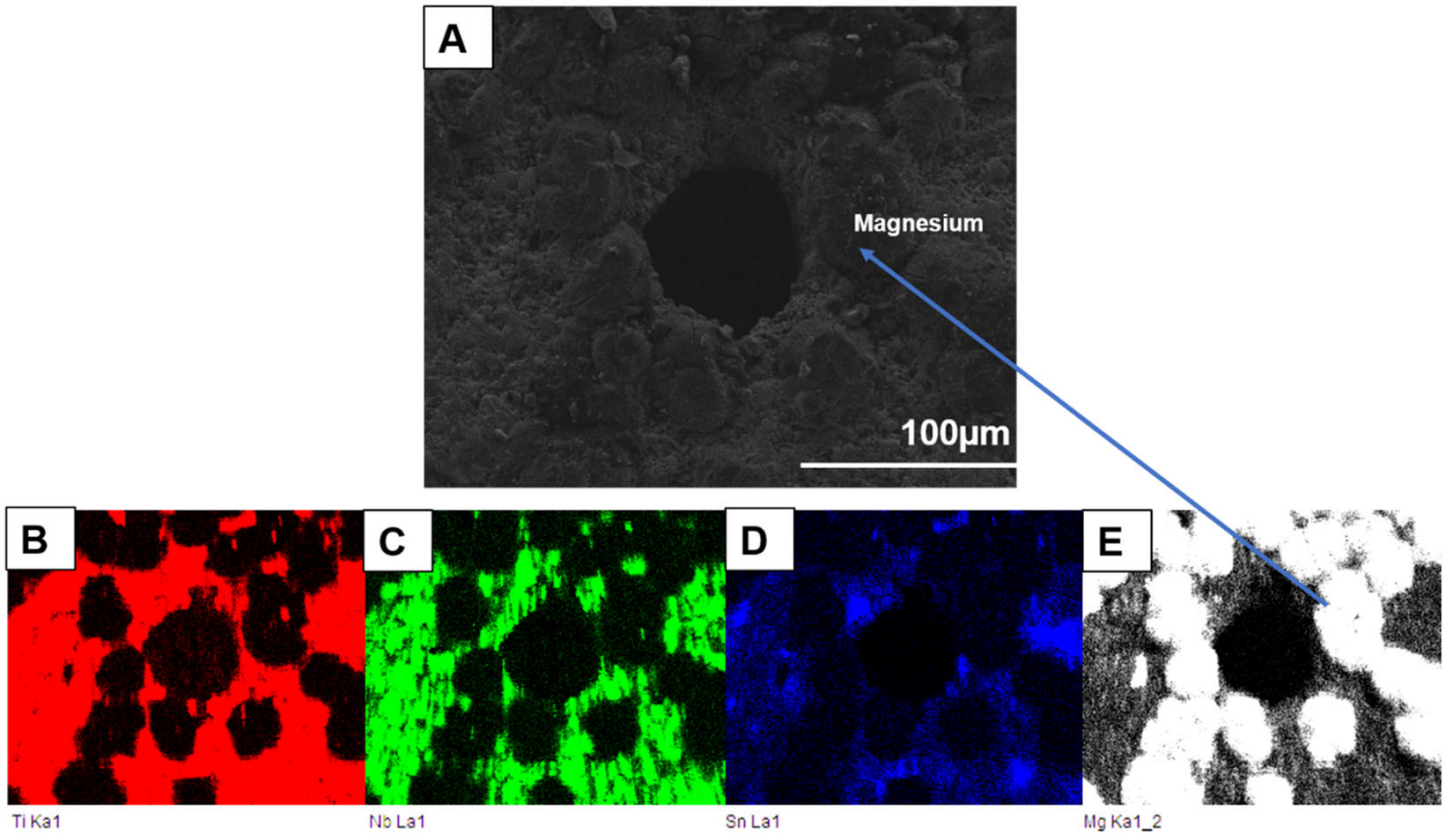
**A** Typical SEM micrograph of TiNbSn alloy (**B**–**E**) elemental map of Ti, Nb, Sn and Mg by EDS detector. ×1000 of magnification

**Fig. 2:**
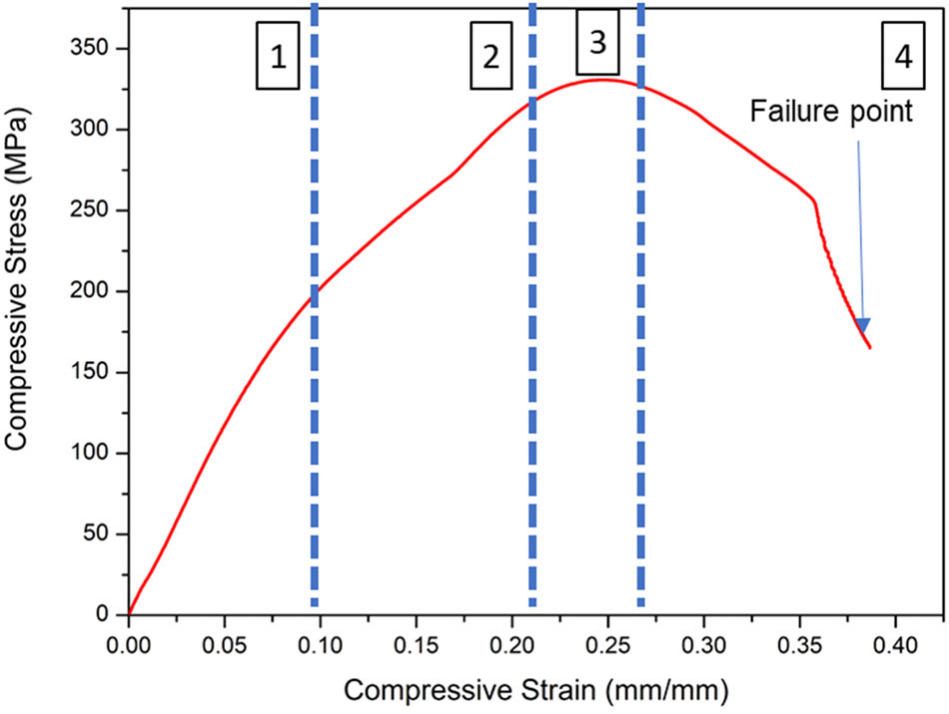
**A** Typical compressive stress versus compressive strain of the Ti-Nb-Sn/Mg alloy with four different deformation zones

The composition profile of the porous alloy is illustrated in Fig. 1A-F. The biomaterial micrograph shows pore formation with a circular morphology of ~100 µm (Fig. 1A). The constituent elements of the alloy (Nb and Sn) and the base metal Ti (Fig. 1B, C and D) are well distributed even around the pores. The three constituent elements (indicated in red, green and blue) present good homogeneity, and there are few regions where one element is more concentrated than another, except for the Mg particles indicated in Fig. 1E, which present low solubility in Ti and in the alloy elements used. Therefore, it can be concentrated in some regions, as seen around the pores, confirming partial evaporation during heating. The mechanical properties of the alloys obtained are indicated in Fig. 2.

Figure 2 presents the typical compressive stress–strain curve of Ti-Nb-Sn/Mg obtained by destructive testing. The curve can be divided into four zones indicated as 1, 2, 3, and 4. In zone 1, the material is elastically compressed up to approximately the proportional limit at the beginning of deformation. The relation between stress and strain is linear, and the slope of the curve is called the elastic modulus (E). In the elastic range, all changes are reversible, i.e., if the stress is removed, the samples return to the original strain. Zone 2 indicates the range where the slope of the curve changes and the plastic region begins. After this point, the sample begins to experience destructive changes (irreversible deformations) represented by zone 3. After the plastic zone, initial failure occurs, and the stress disappears. The location of the breakdown is called the failure/fracture point indicated by the arrow in zone 4.

**Fig. 3:**
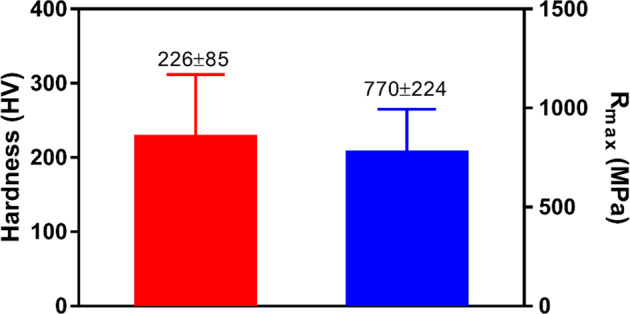
Hardness and Maximum strength evaluation by Vickers

The hardness, strength and elastic modulus were also evaluated by the Rockwell method, compressive test and impulse excitation method. Figure 3 shows the hardness and Str values of the TiNbSn/Mg alloy. The elastic modulus obtained was around of 16 GPa ± 0.2 GPa.

### Direct contact adhesion test

**Fig. 4:**
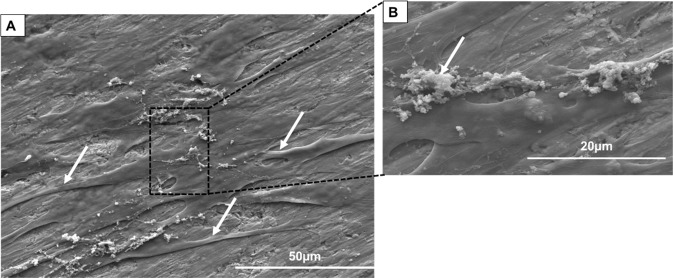
**A** SEM micrograph of BMMSCs adhered to the surface of TiNbSn/Mg using an SE detector. ×2000 of magnification. The white arrows indicate the presence of cytoplasmic projections. **B** SEM micrograph zoom of Fig. 4A indicating possible extracellular vesicle release. ×5000 of magnification

Figure 4A, B shows the presence of eBMMSCs adhered to the surface of the biomaterial after 24 h of incubation. Typical fibroblast/fusiform morphology can be seen. They are also well distributed on the surface of the alloy, presenting cytoplasmic projections, indicated by the white arrows (Fig. 4A). It is also noted that they are well spread out covering substantially the entire alloy surface, making it difficult to differentiate where the cells are adhered and where the biomaterial is due to its good adhesion and integration with the metallic surface. Figure 4B shows in more detail possible extracellular vesicles released by the eBMMSCs (microspheres in brightest contrast indicated by the white arrow).

**Fig. 5:**
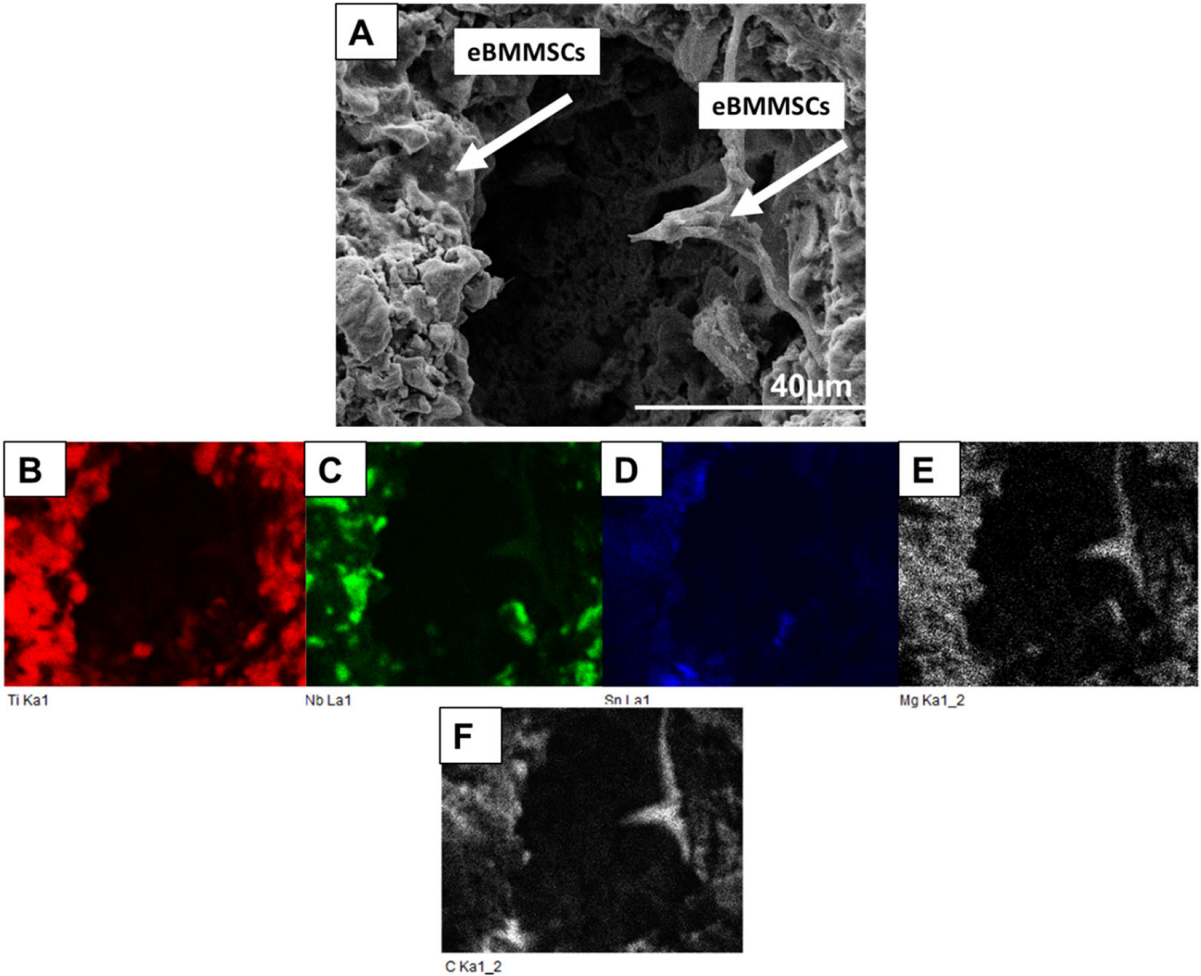
**A** SEM micrograph of Ti-Nb-Sn alloy with eBMMSCs on the surface (**B**–**F**) elemental map of Ti, Nb, Sn, Mg and C by EDS detector. ×3000 magnification

Figure 5 shows the SEM micrograph of eBMMSCs adhered in the region close to and inside the pores. Once again, it is difficult to distinguish the material surface and the cells due to the good adhesion process. Typical fusiform morphology and cytoplasmic projection (indicated by the white arrow) were observed. The Ti, Nb and Sn elements were confirmed by the elemental map obtained in Fig. 5B, C and D. The presence of carbon was detected (Fig. 5F), confirming the presence of eBMMSCs. The carbon enriched zone matched that of magnesium.

### Osteogenic differentiation assay by indirect contact

**Fig. 6:**
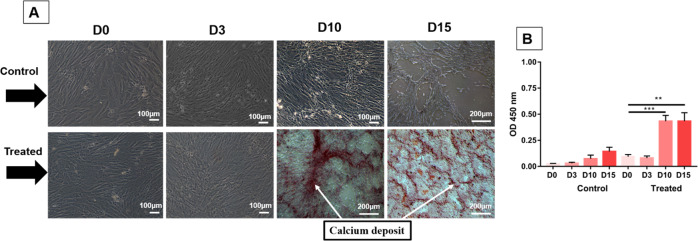
Evolution of osteogenic differentiation by the alizarin red staining assay on days D0, D3, D10 and D15. **A** First line: cells treated with conventional medium. Second line: cells treated with conditioned medium. **B** ALP activity on days D0, D3, D10, D15. *** indicate *P* = 0.006 and ** indicate *P* = 0.002

To evaluate the influence of conditioned medium on matrix mineralization, eBMMSCs were treated with two different media and analyzed after 0, 3, 10, and 15 days, as indicated in Fig. 6A-B. Initially (D0), both cells of the control and treated groups were undifferentiated, as there was no change in the fusiform shape. In addition, they adhere to form a set of homogeneous fibroblast cells. During D3, cells with high growth are noted compared to D0. When they reached D10, the control group continued to proliferate with a fusiform shape. This situation continues until D15, proliferates and shows no change in morphology. The cells treated with conditioned medium, after 10 days (D10), started osteogenic differentiation. The morphology changes can be confirmed by the fusiform shape to polygonal and by the calcium deposit by extracellular matrix. Furthermore, after 15 days (D15), differentiation was more evident due to its polygonal shape and the significant presence of calcium deposits. In Fig. 6B the ALP activity during experimental times D0, D3, D10 and D15 is indicated. Compared to baseline (D0), in the treated group, there was a significant increase in ALP activity at times D10 and D15.The ionic quantification of Mg was performed by absorption spectroscopy of hardened plasma coupling, and the value was 2.2 µg/ml ± 0.3 µg/ml or 0.9 mM ± 0.12 mM, confirming the release and presence of Mg dissolving into the medium.

### Release of reactive oxygen species (ROS)

**Fig. 7:**
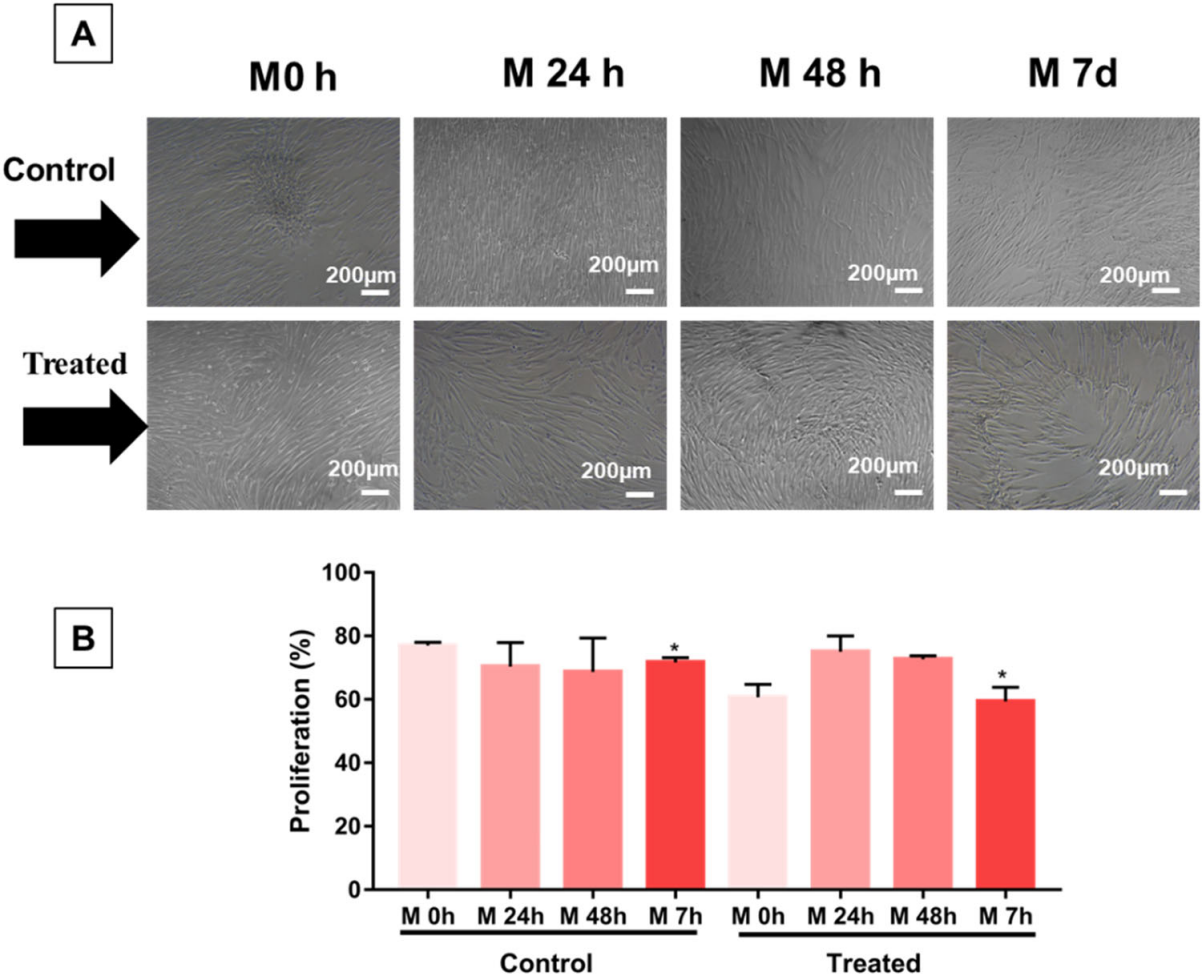
Micrographs of eBMMSCs plated in culture plate wells where they received the treatments. **A** First line: cells treated with conventional medium. Second line: cells treated with conditioned medium with biomaterial. **B** Evaluation of eBMMSC proliferation during the experimental time. * indicates *P* < 0.05

**Fig. 8:**
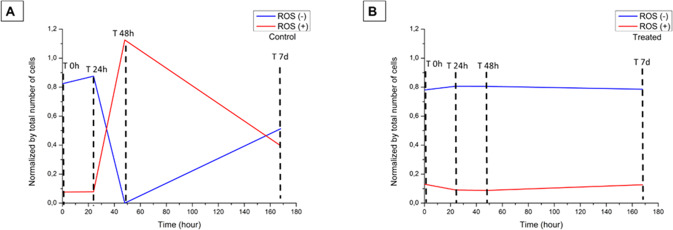
Variability of the eBMMSCs ROS ( + ) and (−) during the respective moments: T 0 h, T 24 h, T 48 h and T 7d. **A** BMMSCs ROS ( + ) and ROS (−) detected in the control group. **B** eBMMSCs ROS (+) and ROS (−) detected in the treated group

To evaluate the release of intracellular ROS during the treatment of the eBMMSCs with the conditioned medium with biomaterial, the experiment was performed in four moments, and during these moments, the eBMMSCs ROS (+) and (+) were quantified. Figure 7A shows the micrographs of eBMMSCs seeded on the wells during the ROS detection experiment. In the control group, there was a set of cells with a fusiform shape, from M 0 h to M 7 d, as well as a proliferation process between these same moments. At 48 h and 7 d, there was a decrease in the number of eBMMSCs. For the treated group, there was an oscillation in the number of adhered cells. At M 0 h, the proliferation was similar to that of the control group. At 24 h, proliferation decreased and grew again at 48 h and decreased at 7 d. Compared to the control group, in the M 7d group, proliferation decreased significantly (P < 0.05). Figure 8 demonstrates the variability of eBMMSCs ROS ( + ) and ROS (−) during the moments M 0 h to M 7d. For the control group, there is a visible oscillation up to M 7 d. For the treated group, eBMMSc ROS ( + ) and (−) remained practically constant until M 7 d. Table 1 shows the values obtained in percentage of eBMMSCs ROS ( + ) and ROS (−) for the control and treated groups.

**Table 1:** Quantification of the % of eBMMSCs ROS (+) and (−)

	Cells/mL	T 0 h	T 24 h	T 48 h	T 7d
Control	ROS (−)	78.16% ± 0.05	85.73% ± 0.03	78.69% ± 0.03	55.03% ± 0.05
	ROS (+)	10.92% ± 0.04	8.36% ± 0.01	10.14% ± 0.01	36.33% ± 0.05
Treated	ROS (−)	78.44% ± 0.00	80.75% ± 0.00	81.45% ± 0.01	79.68% ± 0.01
	ROS (+)	13.26% ± 0.00	9.30% ± 0.00	7.89% ± 0.01	11.14% ± 0.02

First two lines: data related to eBMMSCs treated with conventional culture medium. Last two lines: data related to eBMMSCs treated with conditioned culture medium with biomaterial

**Fig. 9:**
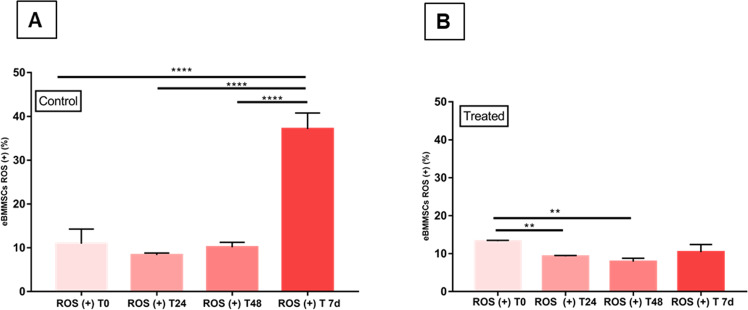
Comparisons of eBMMSC ROS (+) at T 0 h, T 24 h, T 48 h and T 7 days. **A** Comparisons of eBMMSC ROS ( + ) between the time points of the control group. **** indicates *P* < 0.0001. **B** Comparisons of eBMMSC ROS ( + ) between the treatment groups. ** indicates *P* < 0.009 e *P* < 0.0015

Figure 9A, B shows comparisons of the amount of ROS (+) in eBMMSCs in the control and treated groups. In the control group (Fig. 9A) that received conventional culture medium, there was a significant increase (*P* < 0.0001) in ROS (+) in eBMMSCs after 7 d of culture. Until M 48 h, the variation remained practically constant. In the treated group (Fig. 9B), the eBMMSCs ROS (+) significantly decreased (*P* = 0.009, and *P* = 0.0015) after T 24 h and T 48 h, compared to baseline (T 0 h).

## Discussion

The porosity was acquired using Mg powder particles, and during the alloy synthesis process, with heating, these particles were partially evaporated, promoting pore formation in the material. The main spacers used in metallurgy to provide porosity are carbamide, sodium chloride, ammonium hydrogen carbonate and Mg [15–17]. Among these spacers, Mg is still little explored as a spacer. Macropores of ~100 µm were acquired (Fig. 1), and it is believed that pores in the range of 100–400 µm are ideal for bone ingrowth [18]. Other authors claim that size does not directly influence bone formation [18, 19]. Thus, studies related to the development of materials with different pore sizes and their interaction with cells and the in vivo environment are extremely important.

Several studies have reported positive effects of the use of Mg in Ti alloys due to their osteoconductive and biocompatible character [4, 14, 20], but little is known about the oxidative effect of this metal, especially in promising alloys such as Ti-Nb-Sn with a low elastic modulus in mesenchymal stem cells (MSCs).

The mechanical properties acquired for this material were promising in terms of elastic modulus, hardness and compressive strength when compared to materials commercially used in the biomedical sector. In the work by Elias et al., the mechanical properties of commercial Ti grades 2, 4 and 5 were investigated. The elastic moduli values were found in the range of 108 to 115 GPa. Hardness values ranged from 171 to 453 HV, and the Strength ranged from 310 to 932 MPa [21]. For the Ti-Nb-Sn/Mg alloy obtained in the present work, the elastic modulus value was significantly lower (~16 GPa) approaching the bone tissue modulus, which is in the range of 0.5 to 20 GPa depending on the type of bone and from the direction of the analysis [22–24]. The hardness found was 226 HV, which was higher among the values of Ti of different grades studied in the work of Elias et al. [21]. The compressive strength of Ti-Nb-Sn alloy was ~325 MPa, compared to Ti grade 2 (362.6 MPa) [21] and with a value higher than that of cortical bone (41–115 MPa) [25–27].

Figure 2 shows that the BMMSCs fully adhered with a spindle-shaped morphology and sprawled in a short time when in contact with the surface metal. The same was demonstrated in 48 h of cell culture grown on the surface of the alloy with similar chemical composition, suggesting that the obtained alloy has no adverse effects on the cells in question [28, 29]. The interaction of the metal surface with integrins, which are extracellular matrix proteins, promotes better cell adhesion [30]. According to Cavalcanti-Adam, the spreading process is related to the interaction with the metallic surface and the integrins present in the extracellular matrix [31]. The biocompatibility of materials is related to cell behavior in contact with the materials [32]. Cell attachment is presumably the most important stage of cell interaction with a material surface because cell behavior depends on signaling cascades initiated via the adhesion process [33] needed for other cellular activities, such as spreading, proliferation and biosynthesis. After initial attachment, the cells become flattened and finally fully spread [34] Thus, it is clear that the surface of the Ti-Nb-Sn alloy does not provide a harmful surface for eBMMSCs.

Notably, the presence of possible extracellular vesicles or apoptotic bodies released by the eBMMSCs was also notable. Extracellular vesicles tend to be homogeneous in size, between 0.1 and 1 μm, whereas apoptotic bodies are larger, varying from 1 to 5 μm. Such vesicles are considered an additional factor in the mechanism of intercellular communication, allowing cells to exchange proteins, lipids, genetic material and adhesion molecules [35]. Therefore, extracellular vesicles may also have facilitated the adhesion process [36]. According to other studies, extracellular vesicles derived from MSCs and immobilized on Ti surfaces promoted cell proliferation after 3 to 6 days, as shown in Figs. 5 and 6. Recent works have studied the release of apoptotic bodies by MSCs and their impact on bone homeostasis. Apoptotic body treatment is able to ameliorate the osteoporotic phenotype, suggesting the potential use of apoptotic bodies to treat osteoporosis. Apoptotic body treatment directly improves the function of osteogenic cells to enhance bone formation and indirectly inhibits osteoclast activity by upregulating mediators in MSCs related to osteoclast apoptosis [37]. Wang et al. studied the immobilization of extracellular vesicles (exosomes) derived from MSCs adhered to the Ti surface. [38]. These bodies rapidly promote MSC adhesion and proliferation. There are still few studies on the effect of exosomes, microvesicles or apoptotic bodies released by MSCs on biomaterial surfaces. Research in this field demonstrating how the alloying elements, microstructure and roughness of materials influence their increase or decrease can be carried out.

In Fig. 5, the presence of carbon in the elemental map confirms the presence of eBMMSCs, since it is the largest constituent of living matter. The presence of roughness both inside and around the pores may be able to promote bone internal cell growth in the pore region, providing not only anchoring for fixation but also a system capable of allowing stresses to be transferred from the implant to the tissue [39]. In the same figure, note the difficulty of finding the adhered cells being molded according to the surface on which they were exposed. Many works have found that cells have the ability to mold and modify their geometry depending on the environment [40].

The early differentiation of cells treated with conditioned medium by biomaterial was proven due to the clear morphological change from fibroblastic to polygonal shape before treatment with conventional medium and calcium deposition. As one of the differentiation strategies based on the concept that bioactive biological clues can be added to the implant surface to promote the regenerative processes on its surface, one of the important roles of magnesium is observed, which would be able to stimulate osteoconductivity. Mg affects the activity of alkaline phosphatase (ALP), a marker of early osteogenic differentiation, and its activity improves in the presence of Mg particles. In our work, 2.2 µg/ml or 0.9 mM Mg ions were released in the medium. Studies have shown that high doses can hamper the osteointegrative process. Zhang et al. treated human bone marrow stem cells (hBMSCs) with different concentrations of Mg^2+^, and matrix mineralization was significantly inhibited in osteoinductive medium at concentrations equal to or above 1.3 mM. [41]. Wang et al. demonstrated that concentrations of 1.8 mM and 3.8 mM Mg^2+^ significantly decreased calcium oscillation amplitude or frequency, while increasing the concentration of Mg^2+^ from 0.8 to 1.3 mM exhibited no effect on calcium oscillation frequency [42]. The authors deduced that a high Mg^2+^ environment inhibits matrix mineralization by suppressing the calcium oscillation frequency in hBMSCs.

The effect of the culture medium identified on eBMMSC ROS ( + ) was notable after 7 days of analysis, as indicated in Fig. 9. Naturally, in the control group, the eBMMSCs ROS ( + ) increased. This effect was also demonstrated in the works by Tirza et al. [43]. In the treated group, the number of eBMMSCs ROS ( + ) began to fall within 24 h of treatment with the conditioned medium, up to 48 h of analysis and up to 7 days remained constant. Some authors have reported that low extracellular Mg is linked to an increased generation of ROS in different kinds of cells [44].

Using human endometrial MSCs, Lyublinskaya et al. showed that intracellular basal ROS levels are positively correlated with the proliferative status of cell cultures. In fact, they observed that physiologically relevant levels of ROS are required for the initiation of human MSC proliferation and that low levels of ROS due to antioxidant treatment can block stem cell self-renewal [45].

Oxidative stress is a major factor impairing MSC function, resulting in decreased osteogenesis [46]. It is clear that Mg has an effect on decreasing eBMMSCs (ROS) + and on osteogenic differentiation.

## Conclusion

Biological tests of direct and indirect contact with BMMSCs in Ti-34Nb-6Sn/Mg alloy with an elastic modulus close to bone tissue were performed. The BMMSCs adhered quickly to the surface of the biomaterial, and no harmful effects were seen during the experimental time used. The osteogenic differentiation process was observed between days 10 and 15 with the treatment of BMMMSCs with conditioned medium with the alloy. The BMMSCs decreased ROS production during the first 48 h and remained constant for up to 7 days, indicating a possible antioxidant defense potential of magnesium. It can be concluded that Ti-34Nb-6Sn/Mg does not negatively influence the biological functions of cells and is stimulated by the low content of biodegraded magnesium present in the alloy. In our team, works are being developed regarding the Mg concentration in Ti-34Nb6Sn alloys to study their biological and mechanical properties.
